# Editorial Notes

**Published:** 1883-01

**Authors:** 


### EDITORIAL NOTES.

The "Annals of Anatomy and Surgery," in the few years of its publication, has so far won the confidence of the profession that it is now regarded as one of the most valuable journals issued in this country. The coming volume will contain yet more important features for the special field to which the publication is devoted. We commend it to those of our readers whose tastes lead them to devote special attention to surgery and anatomy.

We are glad to note the appointment of Dr. F. Peterson as Lecturer on Pathology, and Curator of the Museum at the Buffalo Medical College. Dr. Peterson proposes to give special attention to pathology, and will make microscopic examinations of fluids and tumors, and post mortem examinations, when desired, for a reasonable fee. The college is to be congratulated on securing the services of Dr. Peterson, who has studied with the great European masters in this department; and we hope the profession will utilize their morbid specimens by sending them to him. They will then be carefully examined, labeled, and preserved in the college museum.

Many of our readers will be glad to learn that the fifteenth edition of the United States Dispensatory will be ready in January, 1883. The editors are Dr. H. C. Wood, Professor of Materia Medica and Therapeutics in the University of Pennsylvania; Joseph P. Remington, Professor of Pharmacy, and Samuel P. Sadtler, Professor of Chemistry, in the College of Pharmacy of Philadelphia. The revision has occupied about three years, and from the high reputation of its distinguished editors we are sure that it has been in all respects most thorough and complete, embracing the most recent discoveries in Materia Medica, Pharmacy, Chemistry and Therapeutics.

We are in receipt of the December number of *The Electrician*, a valuable publication. In its columns new discoveries and inventions in electricity are described and illustrated in a way to interest, not only the scientific, but the general reader. It contains illustrated articles on "Sporting by Electric Light," "The Prosch Telegraph Key," "The Grescom Motor," "The Fuller Electric Light System," "The d'Arsonval Telephone" and "Electrical Sketches." Also, able papers on "An Excursion in a Torpedo Boat," "On the Manufacture of the Weston Carbon," "The Future Electric Lighting," "On the Gold and Stock Telegraph System," "On the Münich Electrical Exhibition," etc., etc. It is published by Williams & Co., 115 Nassau street, New York, at the low price of one dollar per year.

